# Butyrate Improves Skin/Lung Fibrosis and Intestinal Dysbiosis in Bleomycin-Induced Mouse Models

**DOI:** 10.3390/ijms22052765

**Published:** 2021-03-09

**Authors:** Hee Jin Park, Ok-Yi Jeong, Sung Hak Chun, Yun Hong Cheon, Mingyo Kim, Suhee Kim, Sang-Il Lee

**Affiliations:** 1 Department of Internal Medicine and Institute of Health Science, College of Medicine, Gyeongsang National University and Hospital, Jinju 52727, Korea; frontier-jini@hanmail.net (H.J.P.); behappy6-6@naver.com (O.-Y.J.); 9909867@hanmail.net (S.H.C.); hong369c@naver.com (Y.H.C.); mingyokim1@gmail.com (M.K.); 2 Department of Convergence Medical Science, Gyeongsang National University, Jinju 52727, Korea

**Keywords:** systemic sclerosis, butyrate, bleomycin, fibrosis, fecal microbiota

## Abstract

Systemic sclerosis (SSc) is an autoimmune disorder characterized by fibrosis of the skin and internal organs. Despite several studies on SSc treatments, effective treatments for SSc are still lacking. Since evidence suggests an association between intestinal microbiota and SSc, we focused on butyrate, which has beneficial effects in autoimmune diseases as a bacterial metabolite. Here, we investigated the therapeutic potential of sodium butyrate (SB) using a bleomycin-induced fibrosis mouse model of SSc and human dermal fibroblasts (HDFs). SB attenuated bleomycin-induced dermal and lung fibrosis in mice. SB influenced fecal microbiota composition (phyla Actinobacteria and Bacteroidetes, genera *Bifidobacterium* and *Ruminococcus_g2*). SB controlled macrophage differentiation in mesenteric lymph nodes, spleen, and bronchoalveolar lavage cells of mice with bleomycin-induced skin fibrosis. Profibrotic and proinflammatory gene expression was suppressed by SB administration in skin. Furthermore, SB inhibited transforming growth factor β1-responsive proinflammatory expression with increased acetylation of histone 3 in HDFs. Subcutaneous SB application had antifibrogenic effects on the skin. Butyrate ameliorated skin and lung fibrosis by improving anti-inflammatory activity in a mouse model of SSc. Butyrate may exhibit indirect and direct anti-fibrogenic action on fibroblasts by regulating macrophage differentiation and inhibition of histone deacetylase 3. These findings suggest butyrate as an SSc treatment.

## 1. Introduction

Systemic sclerosis (SSc) is an autoimmune fibrotic disease affecting skin and internal organs [1]. The adaptive and innate immune system both play the central role in SSc pathogenesis [2]. The dysregulation of immune responses, which contributes to the overproduction of cytokines, chemokines, and adhesive molecules, leads to myofibroblast activation and secretion of extracellular matrix (ECM) components, contributing to fibrogenesis [3]. These molecular targets involved in the pathogenesis of SSc have been proposed for the treatment of SSc [4,5,6]. Although SSc treatments are focused on managing complications and symptoms of SSc, therapeutic effects against pathological processes of SSc have been evaluated in the substantial agents [6,7]. Several drugs including rituximab, nintedanib, and mycophenolate mofetil were effective in treating SSc-associated complications such as interstitial lung disease (ILD) by targeting vascular alteration, inflammation, or fibrosis of SSc [5,6]. Further research is required to develop more effective therapeutics and strategies to resolve the complex pathological processes that drive SSc.

Recently, many studies have reported on the importance of intestinal microbiota in the pathogenesis of systemic autoimmune diseases [8]. This relationship with microbiota has also been studied in patients with SSc, as the gastrointestinal tract (GIT) is one of the organs most affected by SSc [1,9,10]. A reduction in butyrate-producing bacteria and an increase in proinflammatory bacteria has been observed in intestinal microbiota of SSc patients [1,11]. This evidence indicated that ameliorating intestinal microbiota may be an alternative treatment for SSc. One attempt at targeting the intestinal microbiome of SSc patients suggested that probiotic (non-pathogenic microbes) treatment could improve SSc-associated GIT symptoms [12]. However, probiotics’ efficacy in SSc has not been clearly proven due to a small study [12] and contradicting results demonstrating an immunoregulatory effect of probiotics without improving GIT symptoms in SSc patients [13]. Prebiotics (nutrients for microbiota) constitute another microbiota-based approach; microbiota-derived metabolites have been introduced for the treatment of autoimmune diseases [14]. Until now, a therapeutic intervention using prebiotics and microbiota-derived metabolites has not been studied in the treatment of SSc. The availability of these prebiotics and metabolites highlights their potential as a treatment for SSc, as they can extensively stimulate beneficial bacteria groups and systemically enhance the immune system [15].

Butyrate is a short-chain fatty acid (SCFA), a metabolite produced by fermentation of indigestible fiber by bacteria in the colon [16]. Butyrate not only helps maintain intestinal health by acting as an energy source for intestinal epithelial cells, but it also controls the development of microbial communities and differentiation of immune cells by mediating host–microbe interaction in the intestines [17]. The immunomodulatory and anti-inflammatory activity of butyrate have been demonstrated at the intestinal and extraintestinal level [18]. Butyrate downregulates the release of proinflammatory cytokines from macrophages and dendritic cells (DCs) [19,20]. It also facilitates differentiation of regulatory T cells (Tregs) [21]. In addition, previous studies suggest that butyrate has a therapeutic effect in various animal models including immune-mediated inflammatory diseases such as rheumatoid arthritis, colitis, and obesity [22,23,24,25,26].

Histone deacetylases (HDACs) play a crucial role in the epigenetic regulation of gene transcription [22]. In vivo studies on mice and humans have demonstrated the critical role of the inhibition of HDACs in ameliorating lung fibrosis [27,28]. SCFA, which are HDAC inhibitors, cause enhancement of anti-inflammatory activity and suppression of inflammatory responses in immune cells [29,30]. Butyrate can also inhibit HDAC1 and 3 [29]. Indeed, butyrate regulates the macrophage differentiation program through HDAC3 inhibition [17] and leads to the downregulation of lipopolysaccharide (LPS)-induced proinflammatory mediators in macrophages [19]. These studies suggest the potential efficacy of butyrate as an HDAC inhibitor in treating fibrotic disorders.

Although the useful effects of butyrate in inflammatory diseases, including autoimmune diseases, have been reported, little is known about the underlying mechanisms of sodium butyrate (SB)’s effects in SSc. We hypothesized that butyrate supplementation can improve the symptoms of SSc by positively influencing immune cells in the intestines or other organs, because the gut of SSc patients exhibits intestinal dysbiosis, such as depletion of butyrate-producing bacteria, which might alter the host–microbe crosstalk and immune response in SSc patients [1,11]. Thus, the aim of this study was to investigate the function and potential mechanism of butyrate in skin and lung fibrosis using a mouse model of SSc. Here, we observed that SB impacted fibrosis by modulating immune responses in a bleomycin (BLM)-induced SSc model. The study provides new insights on the application of various gut-derived metabolites such as butyrate in SSc treatment.

## 2. Results

### 2.1. SB Ameliorates Dermal Fibrosis in a BLM-Induced Skin Fibrosis Mouse Model

**Figure 1:**
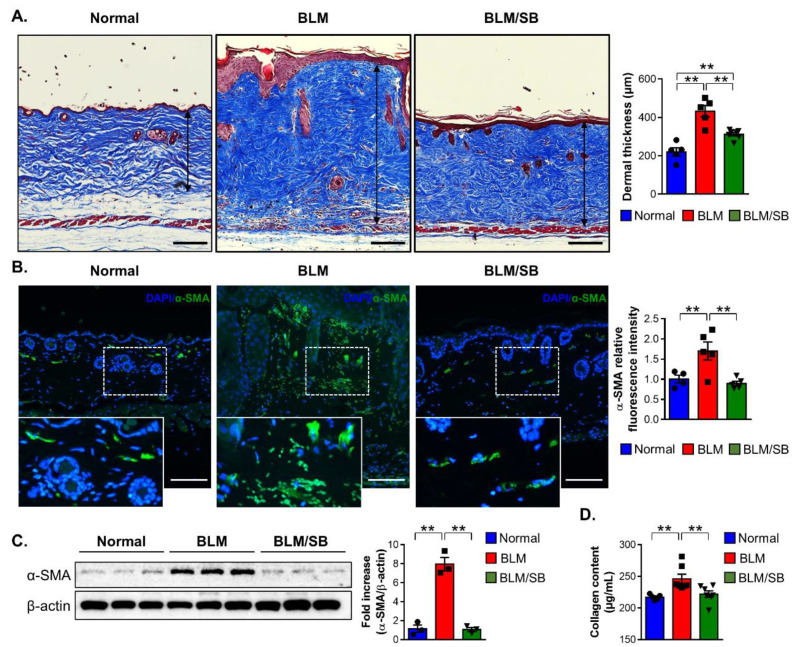
Antifibrotic effects of butyrate in the BLM-induced skin fibrosis mouse model. Bleomycin (BLM) was injected subcutaneously to the back skin of mice five times a week for two weeks. Sodium butyrate (SB) was orally gavaged from two weeks before BLM injection. Skin tissues were then obtained in normal and BLM ± SB mice to evaluate fibrosis. (**A**) Representative images of the Masson’s trichrome stain and dermal thickness (arrow length) in skin tissues. Data are representative of at least four independent experiments with *n* = 5–7/group. (**B**–**D**) Representative immunofluorescence images of skin stained with DAPI (blue) and α-SMA (green) (**B**), representative Western blotting and quantitative analysis of α-SMA expression (**C**), and collagen content (**D**) in the skin. Data are representative of two independent experiments with *n* = 3–5/group; each symbol represents one mouse. Scale bars = 100 µm. DAPI: 4′,6-diamidino-2-phenylindole. ** *p* < 0.01.

To determine whether SB can clinically alleviate skin fibrosis, a BLM-induced skin fibrosis model was used. Two weeks of BLM treatment led to significantly increased dermal thickness in mice (Figure 1A). The dermal thickening caused by BLM treatment was ameliorated by the oral administration of SB (Figure 1A). Expression of the alpha smooth muscle actin (α-SMA) in the skin, a myofibroblast marker, was decreased by the administration of SB in the BLM-induced mouse model of skin fibrosis (Figure 1B,C). Immunofluorescent staining for α-SMA in the skin showed increased expression in BLM-treated mice, while the expression was suppressed by administration of SB (Figure 1B). Administration of SB also reduced protein expression of α-SMA that was increased by BLM injection in the skin (Figure 1C). Furthermore, the collagen content deposited in BLM-injected skin was significantly decreased following the administration of SB (Figure 1D). Taken together, SB attenuated dermal fibrosis by preventing myofibroblast differentiation and collagen synthesis that were stimulated by BLM treatment.

### 2.2. SB Affects Fecal Microbiota Composition Altered in BLM-Induced Skin Fibrosis Mice

**Figure 2:**
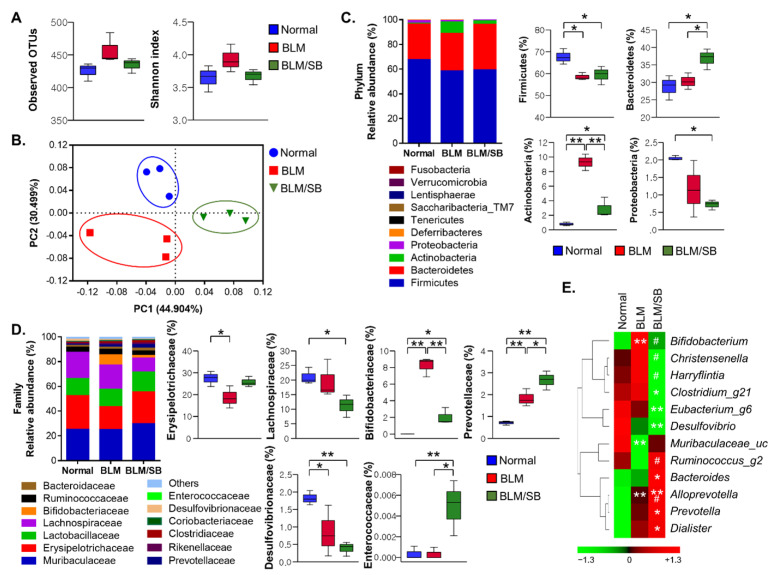
Composition of fecal microbiota. Bleomycin (BLM) was injected subcutaneously for two weeks. Sodium butyrate (SB) was administered orally from two weeks before BLM injection and feces were then obtained. The 16S sequences of fecal microbiota were analyzed in normal and BLM ± SB mice. (**A**) Microbiota richness estimated by the number of operational taxonomic units (OTUs) and Shannon index. (**B**) Microbiota diversity evaluated by principal coordinate analysis plots of UniFrac distances. (**C**,**D**) The overall composition of gut microbiota (left) and statistically significant changes (right) at the phylum level (**C**) and at the family level (**D**). (**E**) Heatmap of differentially abundant microbial genera among groups. Color represents normalized (z-score) relative abundance of bacteria from green (low abundance) to red (high abundance) (**E**). *n* = 3/group; each symbol represents one mouse. * *p* < 0.05, ** *p* < 0.01 in (**C**,**D**). * *p* < 0.05, ** *p* < 0.01 vs. normal, # *p* < 0.05 vs. BLM in (**E**).

The effects of SB on intestinal microbial changes were investigated in the BLM-induced skin fibrosis model using microbial 16S rRNA sequencing. Microbiota species richness (alpha diversity) within each sample was estimated by the number of observed operational taxonomic units (OTUs) and Shannon index in normal, BLM, and BLM/SB groups. The fecal microbiota richness was not statistically different between experimental groups (Figure 2A). However, UniFrac distance-based principal coordinate analysis (PCoA) showed distinct microbial clustering between groups (Figure 2B). It revealed that microbiota profiles were different between the groups.

At the phylum level, BLM resulted in dysbiosis in Firmicutes and Actinobacteria in the gut. BLM treatment significantly reduced the relative abundance of Firmicutes while increasing the relative abundance of Actinobacteria (Figure 2C). Notably, Actinobacteria, which have been enriched by BLM treatment, decreased in abundance after administration of SB. Meanwhile, SB did not restore the relative abundance of Firmicutes decreased by BLM treatment. The frequency of Firmicutes remained at a similarly low level in the BLM/SB group. In addition, Bacteroidetes was more prevalent in the BLM/SB group than in the other groups. Proteobacteria was less prevalent in the BLM/SB group than in the normal control group (Figure 2C).

Next, we undertook a more detailed analysis of bacterial composition at the family and genus levels. The BLM-induced skin fibrosis model showed decreased abundance of Erysipelotrichaceae and Desulfovibrionaceae, while the families Bifidobacteriaceae and Prevotellaceae became more abundant in fecal bacterial composition (Figure 2D). Remarkably, administration of SB reduced the abundance of Bifidobacteriaceae, which belongs to the phylum Actinobacteria that was enriched after BLM treatment. Moreover, the group treated with BLM/SB had a decreased proportion of Lachonospiraceae and an increased proportion of Enterococcaceae compared to those in either the normal or BLM groups, although perturbation of these microbiota was not observed in the BLM group. At the genus level, *Bifidobacterium* was expanded in the BLM group, while it was reduced in the BLM/SB group (Figure 2E). Within the BLM/SB group, *Clostridium_g21*, *Eubacterium_g6*, and *Desulfovibrio* were less abundant compared to those in the normal group, whereas *Bacteroides*, *Prevotella*, and *Dialister* were enriched. *Christensenella* and *Harryflintia* were less abundant, while *Ruminococcus_g2* was more abundant in the BLM/SB group than in the BLM group.

### 2.3. SB Inhibits Infiltration of Inflammatory Monocytes in Mesenteric Lymph Nodes and Spleen of BLM-Induced Skin Fibrosis Mice

**Figure 3:**
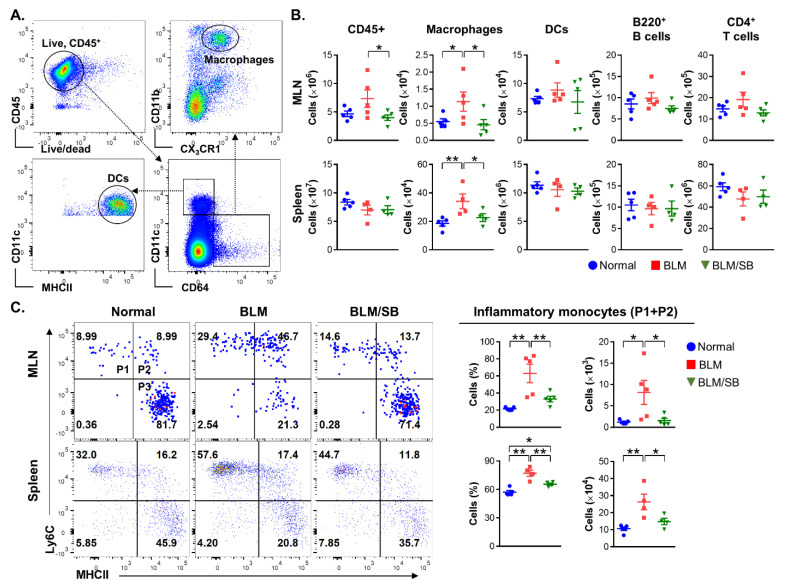
Flow cytometric analysis of immune cell populations in the mesenteric lymph nodes (MLN) and spleen of normal and bleomycin (BLM) ± sodium butyrate (SB) mice. BLM was injected subcutaneously for two weeks. SB was administered orally from two weeks before BLM injection. Immune cells were then obtained from the MLN and spleen. (**A**) Flow cytometric gating strategy for macrophage and DC lineages. (**B**) Flow cytometry was used to assess total leukocytes (CD45^+^), macrophages (CD45^+^CD64^+^CD11b^+^CX_3_CR1^+^), DCs (CD45^+^CD64^−^CD11c^+^MHCII^+^), B cells (B220+), and CD4+ T cells in MLN and spleen from normal and BLM ± SB mice. (**C**) Macrophage lineages were subdivided into phase 1 (P1: newly recruited monocytes), phase 2 (P2: maturing monocytes), and phase 3 (P3: monocyte/macrophage intermediates and resident macrophages) following developmental stages. Inflammatory monocytes were defined as both Ly6C^+^MHCII^−^ (P1) and Ly6C^+^MHCII^+^ (P2). Representative dot plots, percentages (gated on macrophage lineage) and absolute numbers of inflammatory monocytes are shown in the MLN (upper) and spleen (lower) from normal and BLM ± SB mice. Data are representative of two independent experiments with *n* = 4–5/group; each symbol represents one mouse. MHCII: Major histocompatibility complex class II. * *p* < 0.05, ** *p* < 0.01.

Immune cell populations of the mesenteric lymph nodes (MLN) and spleen were analyzed by flow cytometry to investigate the immunomodulatory role of SB in the BLM-induced skin fibrosis model. Gating strategies used to classify macrophage and dendritic cell (DC) lineages are shown in Figure 3A. Administration of BLM caused a significant increase in the number of macrophages (live CD45^+^CD64^+^CD11b^+^CX_3_CR1^+^) in the MLN and spleen. The increased infiltration of macrophages was inhibited by administration of SB. However, BLM treatment did not affect the total number of leukocytes (CD45^+^), DCs (CD45^+^CD64^−^CD11c^+^MHCII^+^), B cells (B220^+^), and CD4^+^ T cells in the MLN and spleen regardless of SB treatment (Figure 3B).

As described above, we observed notable infiltration in the macrophage lineage in BLM-treated mice and blocking of infiltrated macrophages by administration of SB. To further investigate which subsets of macrophages were involved in BLM-induced pathogenesis and the protective mechanism of SB, we divided the macrophage lineage into three subsets that can be classified into phenotypic differentiation stages: phase 1 (P1) was defined as newly recruited monocytes (Ly6C^+^MHCII^−^), phase 2 (P2)—as maturing monocytes (Ly6C^+^MHCII^+^), and phase 3 (P3)—as monocyte/macrophage intermediates and resident macrophages (Ly6C^+^MHCII^−^) (Figure 3C, left panel). SB decreased infiltration of inflammatory monocytes (P1 and P2: CD45^+^CD64^+^CD11b^+^CX_3_CR1^+^Ly6C^+^) accumulated by BLM treatment in the MLN and spleen (Figure 3C). The current study indicates that infiltration of macrophages, particularly of inflammatory monocytes, can play a pathogenic role in BLM-induced skin fibrosis and that this can be blocked by administration of SB. 

### 2.4. SB Prevents Recruitment of Macrophages and Suppresses Upregulation of Profibrotic and Proinflammatory Genes in BLM-Treated Fibrotic Skin

**Figure 4:**
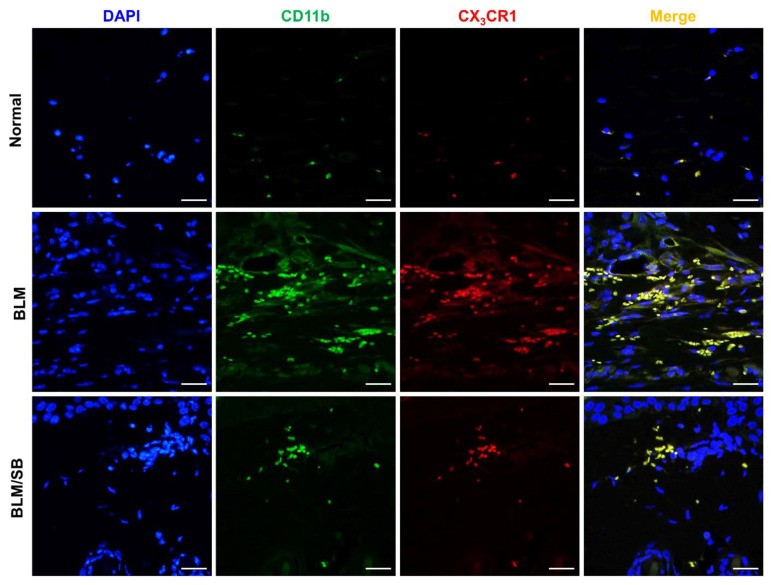
Expression of CD11b^+^CX_3_CR1^+^ macrophages in skin tissues from normal and bleomycin (BLM) ± sodium butyrate (SB) mice. BLM was injected subcutaneously for two weeks. SB was orally administered from two weeks before BLM injection and skin samples were then obtained. Representative immunofluorescent images of skin stained for DAPI (blue), CD11b (green), and CX_3_CR1 (red). Magnification, 400×. Scale bars = 100 µm. *n* = 6/group.

To determine whether BLM and SB affect macrophage infiltration in the skin as observed in the MLN and spleen, we analyzed the number of macrophages by immunofluorescence of CD11b and CX_3_CR1 in skin sections. Immunofluorescent analysis revealed that the number of macrophages (CD11b^+^CX_3_CR1^+^) was increased in the skin of BLM-treated mice (Figure 4), while the increased infiltration of macrophages in the skin was remarkably suppressed by the administration of SB (Figure 4).

**Figure 5:**
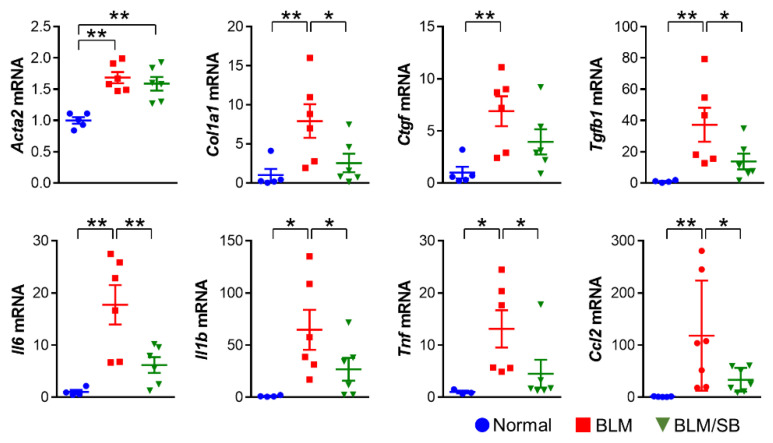
Expression profiles of profibrotic and proinflammatory genes in skin from normal and bleomycin (BLM) ± sodium butyrate (SB) mice. BLM was injected subcutaneously for two weeks. SB was administered orally from two weeks before BLM injection and skin samples were then obtained. *n* = 4–6/group; each symbol represents one mouse. * *p* < 0.05, ** *p* < 0.01.

Next, we explored the expression profiles of mRNAs for profibrotic and proinflammatory mediators in skin. We measured expression levels of α-SMA (*Acta2*), collagen, type I, alpha 1 chain (*Col1a1*), connective tissue growth factor (*Ctgf*), TGF-β1 (*Tgfb1*), interleukin-6 (*Il6*), Il1β (*Il1b*), tumor necrosis factor alpha (*Tnf*), and chemokine (C–C motif) ligand-2 (*Ccl2*) in the skin to compare the gene expressions between groups. BLM treatment upregulated the expression of all the genes listed in Figure 5. Among the genes upregulated by BLM treatment, several profibrotic (*Col1a1*, *Ctgf*, and *Tgfb1*) and pro-inflammatory genes (*Il6*, *Il1b*, and *Tnf*) were suppressed by SB. Moreover, expression of the *Ccl2* gene, which stimulates migration and recruitment of immune cells, was significantly suppressed by SB (Figure 5). These results suggest that SB may relieve skin fibrosis by suppressing production of proinflammatory cytokines, which is mediated through the inhibition of macrophage recruitment to the skin in the BLM-induced skin fibrosis mouse model.

### 2.5. SB Treatment Inhibits TGF-β1-Induced Fibrotic Responses in HDFs 

**Figure 6:**
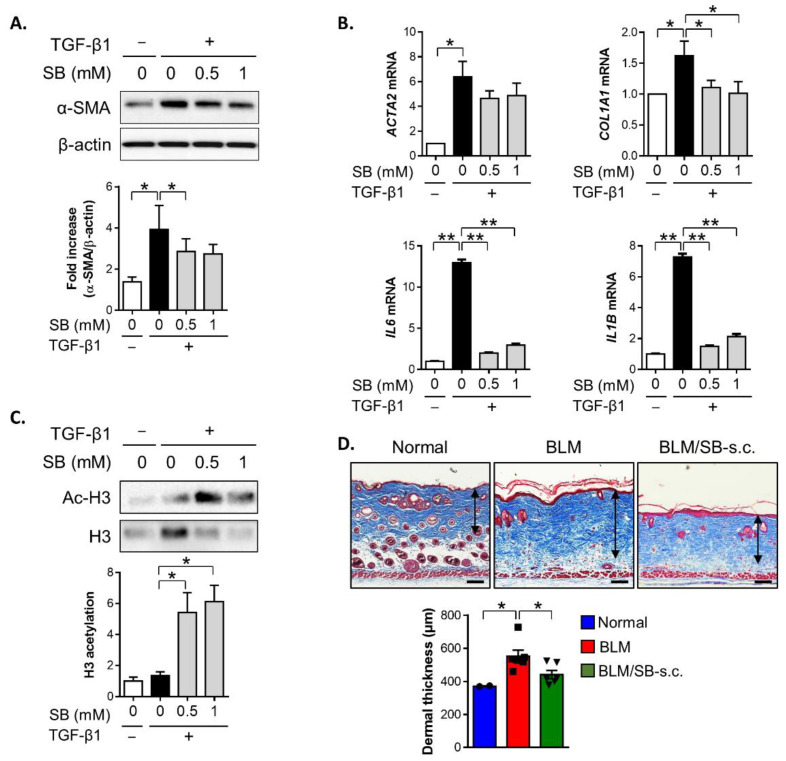
Antifibrotic effect of butyrate on dermal fibroblasts. (**A**–**C**) Primary human dermal fibroblasts (HDFs) were stimulated with TGF-β1 (10 ng/mL) with or without sodium butyrate (SB) (0.5–1 mM) for 24–48 h. Western blotting or qPCR was performed to analyze protein and mRNA expression in the cell lysate from fibroblasts. Representative data of three independent experiments. (**A**) Expression of the α-SMA protein by Western blotting. (**B**) Expression of mRNA for *ACTA2*, *COL1A1*, *IL6*, and *IL1B* by qPCR. (**C**) Acetylated histone H3 (Ac-H3) and histone H3 (H3) protein expression. (**D**) BLM was injected subcutaneously to the back skin of mice five times a week for two weeks with simultaneous subcutaneous (s.c.) injection of SB or phosphate-buffered saline (PBS). Representative images of the Masson’s trichrome stain and dermal thickness in skin sections. *n* = 6/group (*n* = 2 for normal); each symbol represents one mouse. Scale bars = 100 µm, * *p* < 0.05, ** *p* < 0.01.

To clarify the impact of SB treatment on dermal fibroblasts during fibrosis, primary human dermal fibroblasts (HDFs) were stimulated with TGF-β1 at different concentrations of SB in vitro. TGF-β1 stimulation resulted in an increased α-SMA protein expression in HDFs, and the TGF-β1-induced α-SMA protein expression was suppressed by 0.5 mM SB treatment (Figure 6A). The TGF-β1-mediated upregulation of *COL1A1*, *IL6*, and *IL1B* genes in response to TGF-β1 was also remarkably abrogated in the presence of SB in HDFs (Figure 6B). Meanwhile, SB treatment tended to suppress the expression of TGF-β1-induced *ACTA2* gene in HDFs (*p* = 0.11 for 0.5 mM SB, *p* = 0.13 for 1 mM SB versus TGF-β1). Taken together, SB suppressed TGF-β1-responsive profibrotic and proinflammatory activity in dermal fibroblasts.

Previous studies have demonstrated that butyrate exerts antifibrogenic properties by inhibiting HDAC in other cell types. Thus, we assessed acetylation of the histone 3 protein to investigate the mechanism by which SB exerts its antifibrotic effect in dermal fibroblasts. An increase in acetylated histone 3 was observed in TGF-β1-stimulated dermal fibroblasts in the presence of SB compared to those without SB (Figure 6C). These data suggested that SB can exert antifibrotic and anti-inflammatory activity by inhibiting HDAC3 in dermal fibroblasts during fibrogenesis.

To determine whether SB directly contributes to the inhibition of dermal fibrosis in vivo, we attempted to apply SB subcutaneously (s.c.) to the BLM-induced skin fibrosis model. The local BLM and SB injections were co-administered for two weeks, then were evaluated for dermal thickness. The s.c. application of SB reduced dermal fibrosis induced by BLM treatment in mice (Figure 6D). These data indicated that SB has a direct antifibrotic effect on dermal fibroblasts, as evaluated by in vivo and in vitro fibrosis models.

### 2.6. SB Has Anti-Fibrogenic Effects on the Lung in BLM-Induced Fibrosis Mouse Models

**Figure 7:**
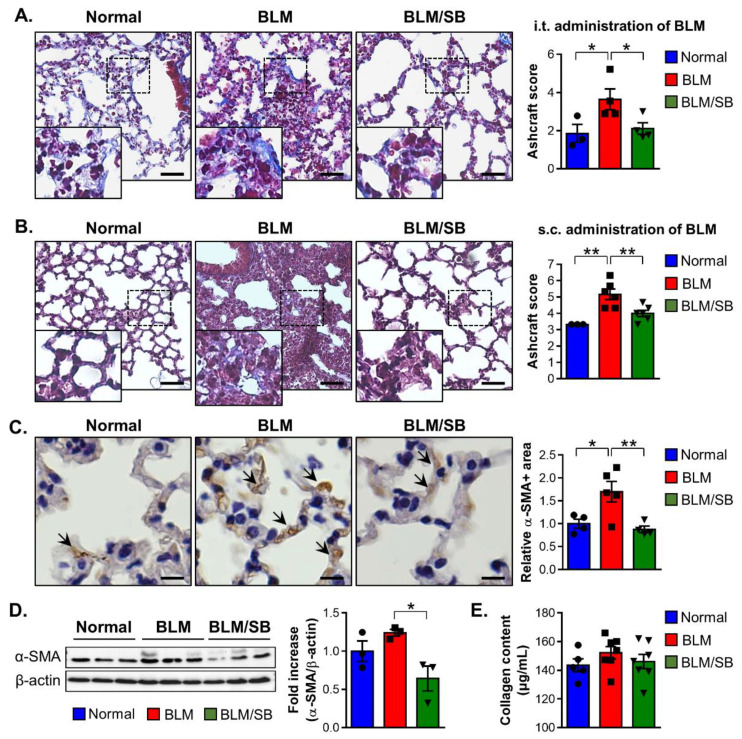
Antifibrotic effects of sodium butyrate (SB) in bleomycin (BLM)-induced lung fibrosis mouse models. (**A**) The BLM-induced pulmonary fibrosis model was used to investigate the effect of SB on lung fibrosis in mice. BLM was administered once intratracheally (i.t.). SB was orally gavaged for five weeks, starting two weeks before BLM injection. Representative images of the Masson’s trichrome stain and histological score (Ashcroft score) in lung tissues from normal and BLM ± SB mice. Scale bars = 500 µm. (**B**–**E**) BLM-induced skin fibrosis model was used to investigate the effect of SB on lung fibrosis in mice. BLM was injected subcutaneously (s.c.) for two weeks. SB was administered orally from two weeks before BLM injection and lung tissues were then obtained. (**B**) Representative images of the Masson’s trichrome stain and Ashcroft score in lung tissues from normal and BLM ± SB mice. The results were verified through four independent experiments. *n* = 5–7/group in each experiment. Scale bars = 500 µm. (**C**) Representative immunohistochemical image of α-SMA (brown colors: arrows). Scale bars = 100 µm. (**D**) Representative Western blotting and quantitative analysis of α-SMA expression. (**E**) Total collagen content in lung tissues from normal and BLM ± SB mice. *n* = 3–6/group; each symbol represents one mouse. * *p* < 0.05, ** *p* < 0.01.

The BLM-induced skin fibrosis model accompanies lung fibrosis in mice. Therefore, to explore antifibrotic effect of SB on the lung, we used two models as follows: pulmonary fibrosis models induced by intratracheal (i.t.) or s.c. injection of BLM. In the two models, administration of SB alleviated lung fibrosis (Figure 7A,B).

The antifibrotic effect of SB on lung fibrosis was further studied in a BLM-induced skin fibrosis model, which has been reported to be an inducible representative experimental model of SSc. Immunohistochemical staining of the lungs revealed a significant increase in the α-SMA^+^ area after BLM treatment, while SB decreased the α-SMA^+^ area against BLM treatment (Figure 7C). However, Western blotting of the lung tissue showed BLM-induced skin fibrosis did not cause elevation of α-SMA (Figure 7D). Although BLM treatment did not lead to a significant change in the protein expression of α-SMA in the lung compared to that in the normal control group, the expression of α-SMA was suppressed in the lung of the BLM/SB group compared to that in the BLM group (Figure 7D). These analyses revealed that SB could inhibit the expression of the α-SMA protein in fibrotic lung tissue. There were no significant differences in the collagen content of the lung between experimental groups (Figure 7E). Taken together, SB could attenuate lung fibrosis by preventing histological modifications and myofibroblast activation of lung tissue cause by BLM-induced skin fibrosis model.

### 2.7. SB Controls Macrophage Differentiation in Bronchoalveolar Lavage Fluid

**Figure 8:**
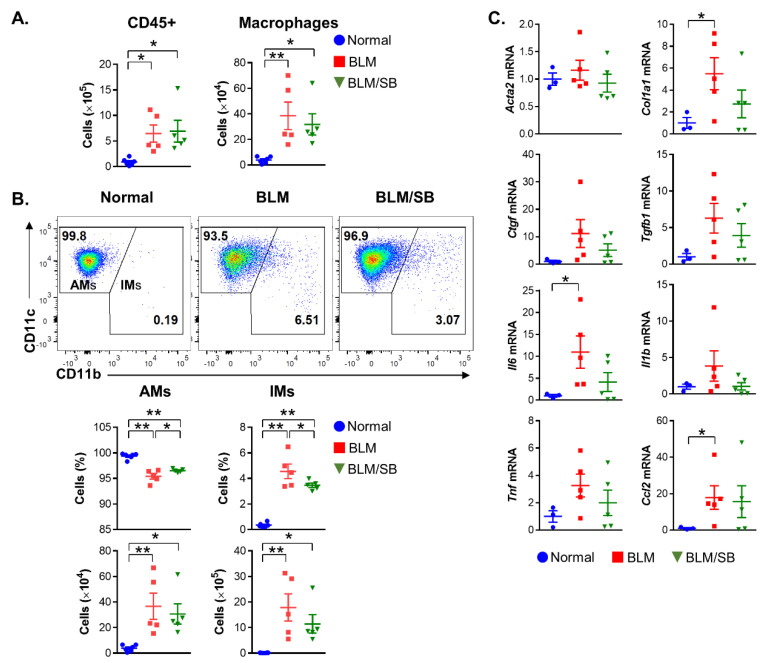
Modulation of macrophage populations in the bronchoalveolar lavage fluid (BALF). (**A**,**B**) Flow cytometry was used to assess macrophage subsets in the BALF from normal and bleomycin (BLM) ± sodium butyrate (SB) mice in the BLM-induced skin fibrosis model. (**A**) Total leukocytes (CD45^+^) and macrophages (CD45^+^CD64^+^F4/80^+^) in the BALF from normal and BLM ± SB mice. (**B**) LMs were divided into AMs (CD11c^+^CD11b^−^) and IMs (CD11b^+^). Representative dot plots, percentages (gated on macrophages), and absolute numbers of AMs and IMs are shown in the BALF from normal and BLM ± SB mice. (**C**) Expression levels of mRNA of profibrotic and proinflammatory mediators in lung tissues from normal and BLM ± SB mice. *n* = 3–5/group; each symbol represents one mouse. * *p* < 0.05, ** *p* < 0.01.

Here, we observed the suppressive effect of SB on infiltrated macrophages in the lymphoid organs and skin of BLM-induced skin fibrosis mice (Figure 3 and Figure 4). Therefore, changes in macrophage populations were also analyzed in the bronchoalveolar lavage fluid (BALF) to investigate the effect of SB on lung macrophages (LMs) in relation to lung fibrosis in the BLM-induced skin fibrosis model. BLM treatment caused infiltration of total leukocytes and macrophages in the BALF. However, SB did not prevent the infiltration of the immune cells in the BALF (Figure 8A).

LMs are mainly composed of alveolar macrophages (AMs) and interstitial macrophages (IMs). To identify specific LM subsets associated with lung fibrosis in BLM-induced skin fibrosis mice and the role of SB in the LM subsets, we further studied LM subsets in the BALF (Figure 8B). The proportion of AMs and IMs was modulated in the BALF of the BLM group, with a decrease in AMs and an increase in IMs (Figure 8B). Meanwhile, SB restored the proportion of AMs and prevented the differentiation toward IMs in the BALF compared to that in the BLM group. However, AM and IM counts did not significantly differ between BLM and BLM/SB groups. Taken together, SB regulated the imbalance of LM subsets in the BALF, but did not prevent total infiltration of LMs in the BALF during lung fibrosis.

Gene expression of *Col1a1*, *Il6*, and *Ccl2* was significantly elevated in the lung tissue of BLM-induced skin fibrosis mice (Figure 8C). Although SB did not significantly suppress the gene expression in the lung tissue, there was some suppression of *Col1a1* (*p* = 0.10) and *Il6* (*p* = 0.10) genes by SB in the lung (Figure 8C).

## 3. Discussion

Our present results indicate that macrophages are a major contributor to fibrosis in the BLM-induced skin fibrosis mouse model. A prominent increase in macrophages was observed in systemic organs such as MLN and the spleen, and target organs such as the skin, as well as the BALF in the BLM-induced skin fibrosis model. SB had a protective effect against the accumulation of inflammatory macrophage subsets in the MLN and spleen, attenuating dermal and lung fibrosis in a mouse model of SSc, making this the first study to report the immunomodulatory activity of SB in an SSc model. SB’s ability to systematically suppress proinflammatory responses means it may be an attractive therapeutic option for SSc.

The BLM-induced skin fibrosis model is a well-established animal model which mimics the early stages of SSc with its accompanying inflammatory changes [31]. Therefore, the BLM model is used to test the anti-inflammatory and antifibrotic efficacy of new drugs. SB clearly exhibited both anti-inflammatory and antifibrotic action during the BLM-induced fibrosis process in our in vivo and in vitro data. Several studies have demonstrated the antifibrogenic effects of butyrate in an idiopathic pulmonary fibrosis (IPF) rat model and in human pterygium and lung fibroblasts [16,32,33]. However, further studies are required to clarify the utility of SB in treating the various processes that make up the pathology of SSc, because SSc is a complex disease with multiple pathologic phenotypes. To confirm whether SB has an antifibrotic effect in other skin fibrosis models besides the BLM-induced fibrosis model, we conducted an experiment using an LPS-induced skin fibrosis model, which has been demonstrated to be a model of early-phase SSc in a previous report [34]. SB decreased the dermal thickness in an LPS-induced skin fibrosis model (Figure S1). Using other SSc models (e.g., tight skin 1 mice) that are more similar to the later stages of SSc without inflammation will provide better understanding of the availability and mechanism of SB in both the later fibrotic stage and early inflammatory stages of SSc [31].

While no studies have reported on fecal microbial composition of BLM-induced skin fibrosis mice, several studies have addressed imbalances in gut microbial composition in patients with SSc. The gut microbial composition of BLM-treated mice was somewhat inconsistent with the gut microbial characteristics observed in SSc patients. A decreased abundance of Bacteroidetes and an increased abundance of Firmicutes has been found in the gut microbiota of SSc patients [11,35,36]. In contrast, gut microbiota of BLM-treated mice showed a decrease in Firmicutes without change in Bacteroidetes in the current study. One of the characteristics of gut microbiota in SSc patients was a reduction of butyrate-producing bacteria (*Roseburia, Clostridium,* and *Ruminococcus*) [1,11]. Unfortunately, we were unable to determine changes in butyrate-producing bacteria beyond the genus level because of the low abundance of the representative butyrate-producing bacteria (*Faecalibacterium* and *Roseburia*) and high proportion of unidentified genera (42.3%) in fecal samples. However, the decrease in Firmicutes in the BLM group supported the idea that the BLM model may be associated with low abundance in butyrate-producing bacteria because Firmicutes is the main butyrate-producing phylum in the human colon [37]. Furthermore, we observed an increase in Bacteroidetes in the gut of SB-supplemented mice, which is a bacterial phylum known to produce acetates and propionates of SCFAs [38]. SB supplementation may influence the colonization of SCFA-producing gut microbiota in the BLM model. However, it is unclear whether SB directly acts on the microbiota composition in the large intestine. Studies have shown that butyrate is quickly absorbed and metabolized in the upper GIT, indicating that the majority does not reach the cecum or colon [39]. Therefore, the antifibrotic action may be achieved through systemic effect by absorption of SB into blood or adjacent tissue (extraintestinal effect) rather than by the intraintestinal effect. Further studies will be required to determine the intestinal effect of SB through a measurement of various SCFA concentrations including butyrate in the large intestine after oral administration of butyrate in an SSc animal model.

Surprisingly, the gut microbiome of BLM-injected mice showed marked expansion of Actinobacteria, which is a rare taxon among the gut microbiota of normal mice [40]. Notably, *Bifidobacterium,* a genus belonging to Actinobacteria, was greatly enriched in BLM-injected mice compared to that in normal mice. Consistent with our finding, an increased abundance of *Bifidobacterium* was reported in the gut of SSc patients [9]. However, *Bifidobacterium* species are widely used as probiotics. A decrease in *Bifidobacterium* species has been correlated with disorders such as inflammatory bowel disease (IBD) [37]. In contrast with other disorders, the reasons for the relatively high abundance of *Bifidobacterium* in patients and animal models of SSc remain unexplained. Since SSc is a multistage disease with a diverse range of phenotypes, it may have unique differences in its microbial community compared to that in other autoimmune diseases [9]. Clinicians should carefully consider the application of commonly used probiotics in SSc patients. When applying *Bifidobacterium* species as probiotics for SSc, finding the specific *Bifidobacterium* species that have been reduced in SSc will be a priority.

Under BLM-induced skin fibrosis, SB treatment led to modulated abundance of several gut bacteria, showing a more abundant commensal phylum (Bacteroidetes) and a less abundant pathobiont phylum (Proteobacteria) than those in the BLM or control groups. Interestingly, SB intervention prevented perturbation of Actinobacteria, particularly *Bifidobacterium*, in BLM-treated mice. However, it remains unclear whether microbiota altered by BLM-induced skin fibrosis and SB intervention are associated with development of or protection from disease. Future research may be required to clarify the pathogenic or therapeutic role of intestinal microbiota that were modulated in the mouse model of SSc and its treated groups.

There are complex mechanisms leading to fibrosis in SSc, but previous results suggested that monocytes and macrophages play a crucial role in skin and lung fibrosis [41]. Early histopathological studies displayed infiltration of macrophages in dermal perivascular lesions of early diffuse SSc skin [42] and the lung interstitium of SSc-associated ILD [43]. Monocytes from patients with SSc-ILD showed enhanced migratory and chemotactic capacity with increased expression of CC-chemokine receptor 1 (CCR1) and CCR2 [41]. Circulating monocytes from SSc-ILD patients showed fibrogenic phenotypes [44], which may contribute to the production of profibrotic cytokines and ECM components, indirectly stimulating fibroblasts [3]. Monocytes can differentiate into various macrophage subsets, which can be further polarized to M1 or M2 macrophages according to microenvironmental stimuli [45]. The macrophages can be a major source of profibrotic cytokines, which contribute to organ fibrosis [3,46]. Under inflammation, Ly6C^+^ inflammatory monocytes can be recruited into inflamed tissue via CCR2–CCL2 signaling, where they are more likely to polarize into inflammatory M1 macrophages, which secrete proinflammatory cytokines such as IL-6 and TNF-α [47]. Another alternative activation state of macrophages is when Ly6C^+^ monocytes lose Ly6C expression, allowing them to polarize to repair tissue as profibrotic M2 macrophages during the resolution stage of inflammation [48].

In a previous study, BLM-treated mice revealed increased infiltration of inflammatory and M2 profibrotic macrophages in the dermis at early (day 7) and late stages (day 21), respectively [49]. Due to the differing activation stages of macrophages following disease duration in the BLM-induced skin fibrosis model, changes in M2 macrophages were not investigated in the present study. This was because the BLM-induced skin fibrosis model was at an early stage of the SSc model (day 14 after BLM injection), which we expect to present as an inflammatory or transitional fibrotic stage rather than as an established fibrotic stage. According to previous studies, infiltration of macrophages was observed in BLM-induced fibrotic skin. Meanwhile, SB treatment blocked the accumulation of macrophages and exerted suppressive efficacy on profibrotic mediators, *Ccl2*, fibroblast activation, and collagen synthesis in the skin. SB might inhibit expression of chemokine receptors associated with monocyte migration, leading to reduced recruitment of monocytes and differentiation of macrophages within the skin. In turn, the decreased accumulation of monocytes and macrophages following SB treatment might alleviate skin fibrosis by suppressing the production of profibrotic factors and fibroblast activation.

CCL2 (known as monocyte chemoattractant protein 1 (MCP-1)) promotes the recruitment of monocytes/macrophages to inflammatory sites [3]. CCL2 expression positively correlates with skin and lung fibrosis in SSc patients [50,51]. Consistent with this, *Ccl2* is considered one of the key factors involved in skin and lung fibrosis of BLM-treated mice. However, the antifibrotic effect of SB on lung tissue does not appear to be mediated through the regulation of *Ccl2*, unlike what is observed in the skin. Additionally, SB did not prevent accumulation of macrophages in the lung tissue observed in BLM treatment.

Based on the results of the current study, we believe SB may control the imbalance of macrophage subsets by affecting differentiation of macrophages to AMs or IMs, which may be associated with an attenuation of lung fibrosis. Pulmonary macrophages divide into AMs and IMs based on their anatomical locations within the lung [52]. AMs reside in the alveolar airspace while IMs are located within the lung parenchyma. Due to these different positions within the lung, most bronchoalveolar lavage (BAL) macrophages are AMs and rarely IMs [53]. Similarly, IMs made up a smaller proportion of BAL macrophages of normal mice. However, IMs were enriched in BLM-treated mice, and the relative percentage of IMs was decreased by SB intervention. IMs are thought to influence pulmonary fibrotic processes in the lung’s interstitial compartment [54]. In BLM-induced IPF mice, IMs showed an M2-like characteristic (CD206^+^) as their profibrotic phenotype during the fibrotic phase (day 21) in the lung [55]. However, there is a lack of data on the emergence and role of IMs in the BAL during fibrosis. IMs, as defined in this study, might be CD11b^+^ infiltrating macrophages—the so-called exudate macrophages—derived from monocytes as described in a previous paper [56]. Increased CD11b^+^ exudate macrophages were reported in the BAL of IPF mice. Further studies should be conducted to clearly discriminate the macrophage subtypes in the BALF.

Butyrate’s inhibitory action on HDACs may be another mechanism by which butyrate alleviates fibrosis, as HDACs regulate gene expression. Recent studies have also suggested that HDAC activity is associated with fibrogenesis. HDAC inhibitors attenuate BLM-induced pulmonary fibrosis by restoring surfactant protein C expression in alveolar epithelial type II cells [57]. Sodium butyrate has been reported to be one of the HDAC inhibitors that has potent anti-inflammatory properties [22]. However, it is not well-known whether SB exerts HDAC inhibitory activity against dermal fibroblasts. One study suggested that butyrate suppresses fibrosis through HDAC inhibitory mechanisms in human pterygium fibroblasts [32]. In agreement with the previous study, SB suppressed TGF-β1-stimulated expression of profibrotic and proinflammatory mediators with increased H3 acetylation in HDFs in vitro. These findings suggest that SB can behave as an HDAC inhibitor against dermal fibroblasts during fibrosis, and consequently can exhibit antifibrotic effects by inhibiting gene transcription involved in fibrogenesis. In addition, butyrate can modulate immune responses of intestinal macrophages via the inhibition of HDAC, which downregulates proinflammatory mediators such as nitric oxide (NO), IL-6, and IL-12 [19]. Therefore, there is also a possibility that SB suppressed fibrosis through HDAC inhibition of both macrophages and fibroblasts in the BLM-induced skin fibrosis model. However, we did not investigate the possibility of HDAC inhibition in macrophages in this study.

SB showed that it might have direct and indirect impacts on skin fibrosis, as evaluated by the oral and s.c. application of SB in the BLM-induced skin fibrosis mouse model (Figure 1A and Figure 6D). Although immune responses to BLM were different between the skin and lung tissue in mice, SB alleviated both skin and lung fibrosis in the BLM models. SB regulated monocyte and macrophage populations in the BLM-induced skin fibrosis model. In addition, SB may suppress proinflammatory and profibrotic mediators via inhibition of HDAC3 on dermal fibroblasts during the fibrosis process. Our study suggested that butyrate may be a promising new candidate for SSc and other fibrosing diseases.

## 4. Materials and Methods

### 4.1. Chemicals and Reagents

Bleomycin (BLM; Dong-A ST, Seoul, Korea) was prepared by reconstitution of the powder with phosphate-buffered saline (PBS) to reach the final concentration of 1 mg/mL. Sodium butyrate (SB; 303410, Sigma-Aldrich, St. Louis, MO, USA) was dissolved in distilled water at the concentration of 50 mg/mL.

### 4.2. Fibrosis Animal Models

Eight- to ten-week-old male C57BL/6 mice were purchased from Central Laboratory Animal Inc. (Seoul, Korea.) All mice were acclimatized under specific pathogen-free conditions (room temperature: 22 ± 2 °C; humidity: 50 ± 10%; 12 h light/dark cycle). Standard rodent chow and drinking water were supplied ad libitum. 

A total of 114 mice were used in this experiment. Mice were randomly divided into three experimental groups: (1) PBS-treated control mice (normal group); (2) skin or lung fibrosis mice induced by s.c. or i.t. injection of BLM (BLM group) (skin fibrosis was induced by s.c. injections of BLM (100 µg/mouse) to a single location on the back five times a week for a total period of two weeks; the induced IPF model was induced by a single i.t. application of BLM (1 U/kg); mice were euthanized three weeks after the BLM application); (3) mice administered SB orally or s.c. in the BLM group (BLM/SB group). More specifically, this group was specified as BLM/SB-s.c. when SB was administered subcutaneously. Oral administration of SB was performed with a 10 mg dose per mouse five times a week over four weeks (five weeks for the IPF model), starting two weeks before BLM injections in BLM-induced scleroderma and IPF models. To evaluate the direct effect of SB on skin fibroblasts and to minimize the systemic effects of SB, s.c. administration of SB was considered during the induction of skin fibrosis. For the study, SB (2 mg per mouse) was administered s.c. five times a week for two weeks, concurrently with a BLM injection in the BLM-induced scleroderma model. No mice died in the experimental groups during the experiment period.

At the end of the experiments, mice were euthanized by intraperitoneal injection of avertin (Sigma-Aldrich). Tissue samples were collected and stored in 10% neutral buffered formalin or at −70 °C until analysis.

### 4.3. Cell Lines, Cell Cultures, and Experimental Design

Primary HDFs (PCS-201-012, ATCC, Gaithersburg, MD, USA) were maintained in the Dulbecco’s modified Eagle’s medium supplemented with 10% fetal bovine serum (FBS), 100 U/mL penicillin, and 100 mg/mL streptomycin under 5% CO_2_ at 37 °C. HDFs were stimulated with 10 ng/mL of human recombinant TGF-β1 (R&D System, Minneapolis, MN, USA) under different concentrations of SB (0, 0.5, or 1 mM) for 24 h and 48 h to analyze gene and protein expression, respectively.

### 4.4. Histological Analysis

Formalin-fixed, paraffin-embedded skin and left lung sections (5 μm) were subjected to Masson’s trichrome staining and observed under microscopic fields. Dermal thickness was measured at six different sites in each skin section for evaluation of skin fibrosis. Morphological fibrotic changes in lung tissue were assessed at three different sites in each lung section according to a modified Ashcroft score [58]. The final score of each section was expressed as an average of scores observed from different sites.

### 4.5. Immunofluorescent and Immunohistochemical Staining

Deparaffinized sections were incubated with primary antibodies overnight at 4 C. Subsequently, the sections were incubated with the secondary antibody after an intense washing step for 2 h at ambient temperature. Coverslips were mounted using the ProLong gold antifade reagent containing DAPI (P36935, Thermo Fisher Scientific, Waltham, MA, USA). The following primary antibodies were used: α-SMA (1 µg/mL, ab7817, Abcam, Cambridge, UK), CD11b (1:200, sc-23937, Santa Cruz Biotechnology, Dallas, TX, USA), and CX_3_CR1 (1:200, sc-377227, Santa Cruz Biotechnology). Secondary antibodies for immunofluorescence were used as follows: anti-mouse Alexa Fluor 488 (1:500, A-21202, Thermo Fisher Scientific), anti-rat Alexa Fluor 488 (1:500, A-21208, Thermo Fisher Scientific), and anti-mouse Alexa Fluor 594 (1:500, A-21203, Thermo Fisher Scientific). Five randomly chosen high-power fields at 400-fold magnification per animal were evaluated and visualized using a Nikon Eclipse Ni-U microscope (Nikon, Tokyo, Japan). Representative images were analyzed for the fluorescent intensity using ImageJ.

Immunohistochemistry was performed on lung sections using a VECTASTAIN Elite ABC Peroxidase Kit (PK-6102, Vector Laboratories, Burlingame, CA, USA). Lung sections were incubated with the α-SMA antibody (0.034 µg/mL, ab7817, Abcam), then with a biotinylated secondary antibody (BA-9200, Vector Laboratories, Burlingame, CA, USA), followed by color development with 3,3′-diaminobenzidine (DAB) systems (D5637, Sigma-Aldrich). The images from slides were acquired using the NIS-Elements software with a Nikon microscope imaging system (Nikon Instruments) at the magnification of ×400. The average percentage of the α-SMA positive area was quantified using the ImageJ software and normalized by the average α-SMA positive area of the control group [59].

### 4.6. Western Blot Analysis

Proteins from tissues and cells were extracted with a RIPA buffer (89900, Thermo Fisher Scientific) containing a protease and phosphatase Inhibitor Cocktail (GenDEPOT, Baker, TX, USA). The protein lysates (20–40 µg) were separated by 12–15% SDS-PAGE and transferred to polyvinylidene fluoride (PVDF) membranes (IPVH00010, Merck Millipore, Darmstadt, Germany). Membranes were blocked in 5% skimmed milk with a Tris-buffered saline containing 0.1% Tween 20 for 1 h and probed with primary antibodies overnight at 4 °C as follows: α-SMA (0.341 µg/mL, ab7817, Abcam), Histone 3 (1:1000, sc-517576, Santa Cruz Biotechnology) and Acetylated Histone 3 (1:1000, sc-56616, Santa Cruz Biotechnology). The membranes were incubated with horseradish peroxidase-conjugated secondary antibodies (1:3000, 1706515, 1706516, Bio-Rad, Hercules, CA, USA) for 1 h. The signal was developed with ECL Western Blotting Substrates (Bio-Rad). The quantification was performed using ImageJ and normalized against β-actin. Meanwhile, acetylation of Histone 3 was normalized against Histone 3. The images of the whole membrane were presented in Figures S2–S4.

### 4.7. Collagen Measurement

The total collagen contents of skin and lung tissue samples were quantified by detecting hydroxyproline using a Quickzyme Total Collagen assay kit (Quickzyme Biosciences, Leiden, The Netherlands) according to the manufacturer’s protocol. The absorbance at 570 nm was detected on a Versamax microplate reader using the SoftMax Pro 6.5.1 software (Molecular Devices, Wokingham, UK).

### 4.8. Fecal Collection and Microbiota Analysis

Fecal pellets were collected under sterile conditions and immediately stored at −70 °C until analysis. Fecal DNA was isolated using a QIAamp DNA Stool Mini Kit (Qiagen, Valencia, CA, USA) according to the manufacturer’s instructions.

Amplicon library preparation was performed based on the Illumina library preparation protocol (Illumina, San Diego, CA, USA). Quantitative real-time PCR (qRT-PCR) was performed to amplify the V4 region of the bacterial 16S rRNA gene using 515F and 806R primers. Amplicons were quantified and quality-checked using a Qbit system (Thermo Fisher Scientific) and an Agilent 2000 Bioanalyzer (Agilent Technologies, Santa Clara, CA, USA). Sequencing was performed using the ISeq100 (Illumina) to produce 300 bp paired-end reads. A total of 852,570 reads were generated, with an average of 94,730 reads per sample.

The reads were then clustered into OTUs using EZBioCloud (ChunLab, Seoul, Korea) at the cutoff value of 97% similarity. Observed out counts and Shannon index were used to analyze microbial richness (α-diversity of species). PCoA was performed on the UniFrac distance matrices to identify microbial diversity among groups.

### 4.9. Cell Isolation and Flow Cytometric Analysis

Single-cell suspensions from MLN and spleen were mechanically homogenized. The single-cell suspensions were filtered through 100 μm cell strainers and washed with RPMI 1640 supplemented with 10% FBS. For the spleen, red blood cells were lysed with an RBC lysis buffer (420301, BioLegend, San Diego, CA, USA) for 5 min. Lysis was stopped and washed by adding sufficient PBS. For collection of the BALF, the trachea was cannulated with a 20-gauge intravenous catheter after ligation of the left main bronchus. Then, the BALF was collected by flushing the airways three times with 1 mL of cold PBS. Approximately 0.8 mL of the BALF was recovered from each collection, which was centrifuged at 500 rpm for 5 min. Single cells from the MLN, spleen, and the BALF were resuspended in PBS supplemented with 5 mM EDTA and 2% FBS. Total cell counts were determined using a TC20 automated cell counter (Bio-Rad).

For flow cytometry analysis, 1 × 10^6^ cells were incubated on ice in 100 μL of a diluted antibody solution for 30 min. The following antibodies were used: MHCII-BV421 (1:1000, clone M5/114.15.2, 562564, BioLegend), FVS510 (1:300, 564406, BD Bioscience, San Jose, CA), Ly-6C-BV605 (1:200, clone AL-21, 563011, BD Bioscience), CD64-BV780 (1:100, clone X54-5/7.1, 741024, BD Bioscience), CD11b-BB515 (1:200, clone M1/70, 564454, BD Bioscience) or CD11b-APC-Cy7 (1:200, clone M1/70, 101226, BioLegend), CD11c-BB700 (1:200, clone HL3, 745899, BD Bioscience), CX_3_CR1-APC (1:200, clone SA011F11, 149008, BioLegend), CD45-APC-Cy7 (1:200, clone 30-F11, 557659, BD Bioscience) or CD45-FITC (1:200, clone 30-F11, 11-0415-85, eBioscience, San Diego, CA), B220-PE-Cy7 (1:300, clone RA3-6B2, 552772, BD Bioscience), CD4-PE-Cy7 (1:300, clone GK1.5, 56-0041-82, eBioscience), and F4/80-PE (1:200, clone BM8, 12-4801-82, eBioscience). Stained samples were analyzed using a LSRFortessa^TM^ X-20 flow cytometry instrument (BD Bioscience). Data were analyzed using FlowJo version 10 (FlowJo, Ashland, OR, USA).

### 4.10. RNA Isolation and Gene Expression Analysis

Total RNA was extracted using the TRIzol™ Reagent (Thermo Fisher Scientific). One microgram of RNA was reverse-transcribed with an iScript cDNA synthesis kit (1708891, Bio-Rad). Real-time PCR was performed using a ViiA 7 Real-time PCR Detection System (Applied Biosystems, CA, USA) with either a Taqman gene expression master mix (4369016, Thermo Fisher Scientific) or a Power SYBR Green PCR master mix (4367659, Thermo Fisher Scientific). Expression of target genes was calculated using the 2^−ΔΔCt^ comparative method for relative quantification after normalization against glyceraldehyde 3-phosphate dehydrogenase (GAPDH).

The following Taqman assays (Thermo Fisher Scientific) were used: mouse (m) *Acta2* (Mm00725412_s1), *mCol1a1* (Mm00801666_g1), *mCtgf* (Mm01192933_g1), *mTgfb1* (Mm01178820_m1), *mIl6* (Mm00446190_m1), *mIl1b* (Mm00434228_m1), *mTnf* (Mm00443260_g1), *mGapdh* (Mm99999915_g1), human (h) *ACTA2* (Hs00426835_g1), *hCOL1A1* (Hs00164004_m1), and *hGAPDH* (Hs07258911_g1). Primer sequences used for SYBR detection are presented in Table S1.

### 4.11. Statistical Analyses

GraphPad Prism version 6 (San Diego, CA, USA) was used for visualization and statistical analysis of data. Data are expressed as the mean ± standard error of mean (SEM). Data were analyzed by one-way analysis of variance (ANOVA) or the Kruskal-Wallis test followed by the Fisher’s least significant difference (LSD) or Mann-Whitney test. *p*-values less than 0.05 were considered statistically significant.

## 5. Conclusions

Sodium butyrate attenuated skin and lung fibrosis in the SSc mouse model. Myofibroblast differentiation was reduced, and anti-inflammatory activity was improved. Fecal microbiota of SB-administered mice exhibited a distinct composition compared to that in the fecal microbiota from the BLM group. Infiltration by monocytes or macrophages and imbalances toward inflammatory macrophages induced by BLM were suppressed by SB intervention. In addition, SB directly downregulated profibrotic and proinflammatory genes via inhibition of HDAC3 in dermal fibroblasts. These results indicate that the antifibrotic efficacy of SB was achieved by multiple mechanisms during tissue fibrogenesis, which include indirect actions on immune cells and a direct action on fibroblasts. Further research is needed to ascertain how these factors are linked to each other, or whether they act independently in alleviating fibrosis. In conclusion, this study suggests that SB is a potential candidate for the treatment of various fibrotic diseases as well as SSc, particularly in its early stages.

## Data Availability

The data presented in this study are available on request from the corresponding author.

## Supplementary Materials

The following are available online at https://www.mdpi.com/1422-0067/22/5/2765/s1.
