# Supplementary material for: Butyrate Improves Skin/Lung Fibrosis and Intestinal Dysbiosis in Bleomycin-Induced Mouse Models
Source: Int J Mol Sci. 2021 Mar 9;22(5):2765. doi: 10.3390/ijms22052765 (PMC7967124; doi:10.3390/ijms22052765)
Supplement: Supplementary file 1 [file ijms-22-02765-s001.pdf]

Supplementary Materials

Table S1. Primer sequences for quantitative real-time PCR.

Species	Gene symbol	Direction	Sequence
Mouse	*Ccl2*	Reverse	5′- CCAGCCTACTCATTGGGAT -3′
		Forward	5′- GGGCCTGCTGTTCACAGTT -3′
Mouse	*Gapdh*	Reverse	5′- ACCCAGAAGACTGTGGATGG -3′
		Forward	5′- ACACATTGGGGGTAGGAACA -3′
Human	*IL6*	Reverse	5′- CCTCAGACATCTCCAGTCCT -3′
		Forward	5′- AATGACGACCTAAGCTGCAC -3′
Human	*IL1b*	Reverse	5′- TACCTGTCCTGCGTGTTGAA -3′
		Forward	5′- TCTTTGGGTAATTTTGGGATCT -3′
Human	*GAPDH*	Reverse	5′- AGTCAGCCGCATCTTCTTTT -3′
		Forward	5′- CCAATACGACCAAATCCGTT -3′

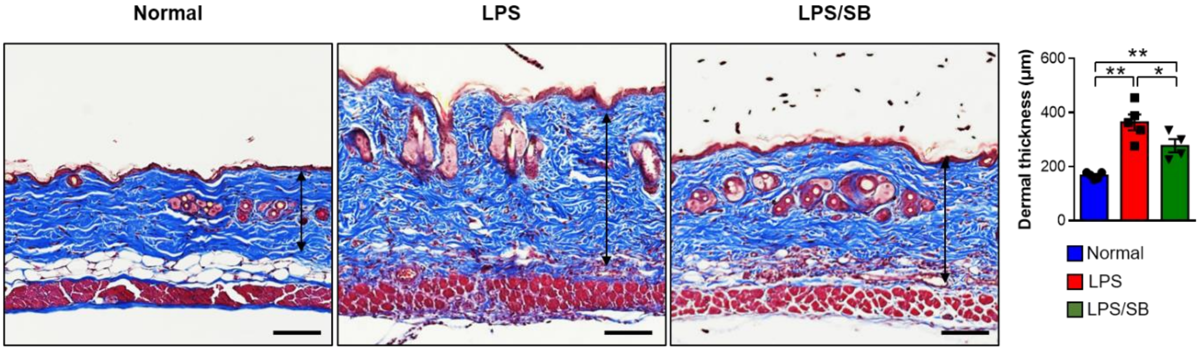

Figure S1. Antifibrotic effect of butyrate in LPS-induced skin fibrosis mouse model. Lipopolysaccharide (LPS) was injected subcutaneously on the back skin of mice five times a week for two weeks (5 μg/mouse). SB was orally gavaged from two weeks before LPS injection. Skin tissues were then obtained in normal and LPS ± SB mice to evaluate skin fibrosis. Representative images of Masson's trichrome stain and dermal thickness in skin tissues from normal and LPS ± SB mice. n = 4–5/group. Scale bars = 100 μm. *$p < 0.05$, **$p < 0.01$.

Uncropped images of the western blot analyses are presented in Figure S2–S4.

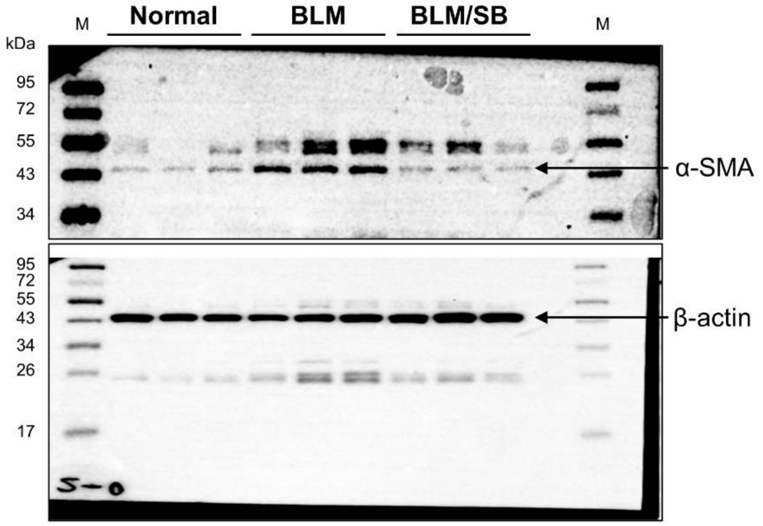

Figure S2. Western blot analysis of α-SMA in skin tissues. BLM was injected subcutaneously on the back skin of mice five times a week for two weeks. SB was orally gavaged from two weeks before BLM injection. Skin tissues were then obtained in normal and BLM ± SB mice. After extracting proteins from skin tissues, western blot was performed to analyze α-SMA protein expression. β-actin was used as a loading control. M: Size marker.

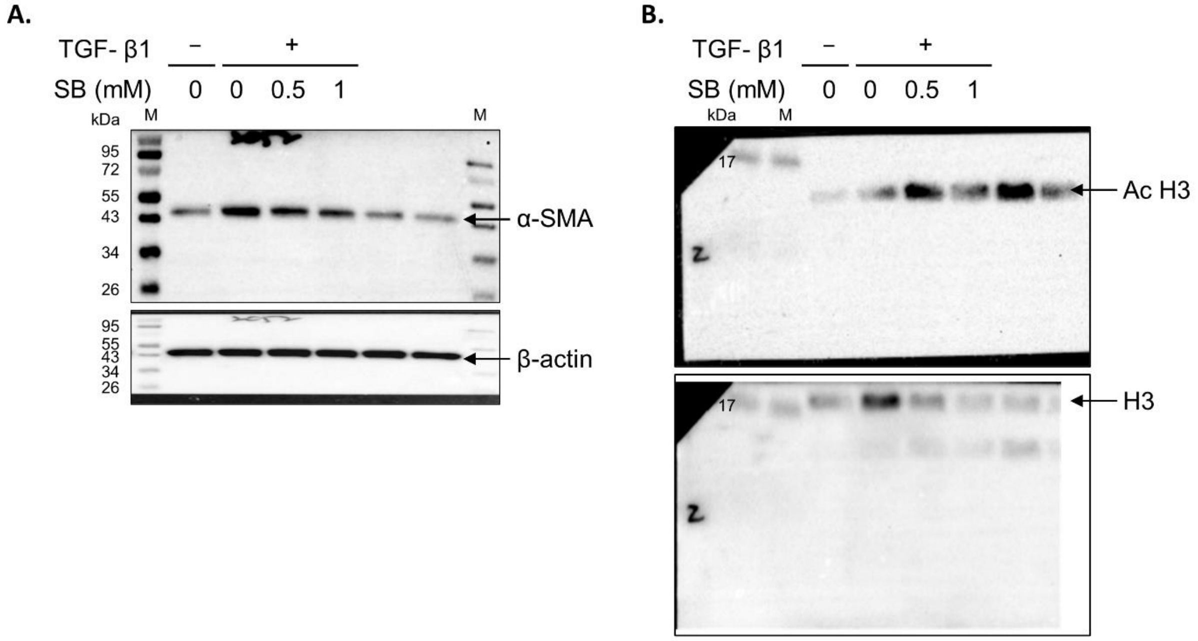

Figure S3. Western blot analysis of α-SMA and Ac H3 in HDFs. Primary HDFs were stimulated with TGF-β1 (10 ng/mL) with or without SB (0.5–1 mM) for 48 h. Western blot was performed to analyze α-SMA (A) and Ac-H3 protein expression (B). β-actin and H3 were used as a loading control for α-SMA and Ac-H3, respectively. M: Size marker.

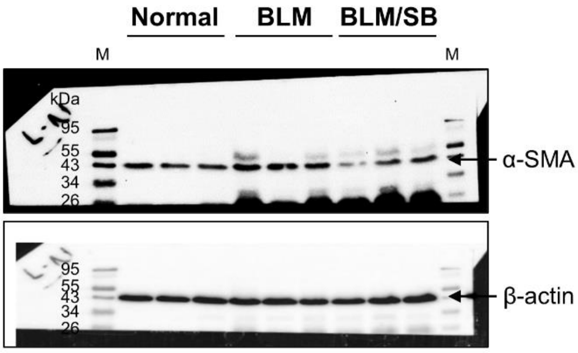

Figure S4. Western blot analysis of α-SMA and β-actin in lung tissues. BLM was injected subcutaneously on the back skin of mice five times a week for two weeks. SB was orally gavaged from two weeks before BLM injection. Lung tissues were then obtained in normal and BLM ± SB mice. After extracting proteins from lung tissues, western blot was performed to analyze α-SMA protein expression. β-actin was used as a loading control. M: Size marker.
